# Resources for assigning MeSH IDs to Japanese medical terms

**DOI:** 10.5808/GI.2019.17.2.e16

**Published:** 2019-06-27

**Authors:** Yuka Tateisi

**Affiliations:** National Bioscience Database Center, Japan Science and Technology Agency, Tokyo 102-8666, Japan

**Keywords:** Japanese language resource, medical vocabulary, MeSH

## Abstract

Medical Subject Headings (MeSH), a medical thesaurus created by the National Library of Medicine (NLM), is a useful resource for natural language processing (NLP). In this article, the current status of the Japanese version of Medical Subject Headings (MeSH) is reviewed. Online investigation found that Japanese-English dictionaries, which assign MeSH information to applicable terms, but use them for NLP, were found to be difficult to access, due to license restrictions. Here, we investigate an open-source Japanese-English glossary as an alternative method for assigning MeSH IDs to Japanese terms, to obtain preliminary data for NLP proof-of-concept.

## Introduction

Tokenization, or word segmentation, is an indispensable first step in natural language processing (NLP). Unlike languages such as English, in which sentences are already segmented into words by spaces, tokenization is not a trivial process in Japanese, where usually no word delimiters are provided.

Systems called morphological analyzers, which tokenize sentences and assign parts of speech to the tokens, are used for this purpose, and most current morphological analyzers require dictionaries. In this process, biomedical text requires special dictionaries, because technical terms often cause problems as out-of-vocabulary terms.

In BLAH5 (Biomedical Linked Annotation Hackathon 5), the current status of Japanese medical vocabularies, especially the Japanese version of Medical Subject Headings (MeSH) is reviewed, and the creation of an open-source alternative is attempted.

## Medical Subject Headings (MeSH)

MeSH [1] is a medical thesaurus developed and maintained by the U.S. National Library of Medicine (NLM) for indexing and cataloging biomedical research articles. MeSH consists of descriptors, qualifiers, and supplementary concept records (SCRs). As of 2018, MeSH contained 28,939 descriptors, 79 qualifiers, and 244,778 SCRs, with each mapped to a unique ID (MeSH ID). Descriptors are hierarchically organized sets of medical concepts, and a descriptor record has a heading (a term in a controlled vocabulary for describing the subjects of articles), entry terms (synonyms to the heading), tree numbers (positions in the hierarchy), scope note (the definition of the subject, in natural language text), and other information. A descriptor may have more than one tree numbers (thus, the MeSH hierarchy is not a tree but a lattice). Qualifiers are terms that are used with a descriptor to define the domain of the study for which the descriptor is applied. SCRs consist mostly of names of substances, having headings and synonyms, but not tree numbers. Instead, SCRs are mapped to the headings in descriptor records.

Due to synonyms, the number of terms that can be mapped to records far exceeds the number of records. For example, there are 234,842 unique terms (headings and synonyms) in MeSH descriptors, and 609,418 unique terms in supplementary concepts. As the synonyms are mapped to headings, via MeSH IDs, and are hierarchically organized, via tree numbers, MeSH represents synonym- and hypernym-hyponym relationships between biomedical terms, and is thus a useful resource for text mining.

MeSH is openly available (downloadable) from the NLM site, and is also included as a part of the Unified Medical Language System (UMLS), which also includes translation of MeSH into other languages, including Japanese. The Japanese translation of MeSH, included in the UMLS, was created by NPO Japan Medical Abstracts Society (JAMAS, *Igaku-Chuo-Zasshi*). The current version, as of February 2019, was released in 2015, and is based on MeSH 2014.

Although the original English MeSH is openly available, other MeSH translations in the UMLS (Czech, Dutch, Finnish, French, German, Italian, Japanese, Latvian, Norwegian, Polish, Portuguese [Brazilian], Russian, Croatian, Spanish, and Swedish) carry the Category 3 License Restriction (https://uts.nlm.nih.gov/license.html); that is, they can only be used at the licencee's site, and cannot be incorporated into publicly accessible computer-based information systems, thus prohibiting creation of open-source, derivative works. These restrictions significantly complicate adapting MeSH for dictionaries used for various NLP tasks.

## Japanese Language Resources with Mappings to MeSH

There are several online resources in Japan which have MeSH information. Unfortunately, none are fully open for NLP. For example, the *Igaku-Chuo-Zasshi* Thesaurus (https://www.jamas.or.jp/database/thesaurus.html), created by JAMAS, organizes Japanese medical terms, based on MeSH tree structures. The latest version (version 9), released in 2019, is freely available for searching and browsing, without registration, but is not downloadable. This thesaurus is based on MeSH 2018, but is not its direct translation. It has 28,247 of 28,939 MeSH 2018 descriptors, and 3,513 terms outside the original MeSH, such as names of drugs sold in Japan and names of Japanese locations and institutions. The terms excluded from MeSH 2018 mainly consist of the names of foreign locations and institutions. The previous version (version 8), released in 2015, was based on MeSH 2014, and claimed to be the Japanese MeSH included in the UMLS. However, while the data download is not free, and the price is not made public.

The Japanese Association of Medical Sciences (JAMS, *Nihon Igakkai*) provides an English-Japanese/Japanese-English medical dictionary online (http://jams.med.or.jp/dic/mdic.html), to registered users, for searching. Download of data, however, is not available. The latest version of the online dictionary (November 2016) has 71,067 Japanese terms and 70,103 English terms, corresponding to 50,088 concepts. The terms in MeSH (and their translation to Japanese) are marked as such, and the top of the MeSH tree numbers (e.g., C14 for *abdominal aortic aneurysm*, whose tree numbers are C14.907.055.239.075 and C14.907.109.139.075) are provided. This MeSH coding is based on MeSH 2014.

The Life Science Dictionary (https://lsd-project.jp/cgi-bin/lsdproj/ejlookup04.pl) [2] is developed and regularly updated at Kyoto University. The dictionary actually consists of three mutually linked components: the English-Japanese/Japanese-English dictionary, the thesaurus, and the corpus. The English-Japanese/Japanese-English dictionary has 117,857 English terms and 132,100 Japanese terms. The entries have been assigned ICD-10 (International Statistical Classification of Diseases, version 10) codes, and a link to the thesaurus, where applicable. The thesaurus is a subset of English terms in the dictionary which have corresponding entries in MeSH (the current thesaurus is based on MeSH 2018), organized in the MeSH hierarchy, with synonyms both in English and Japanese, and related terms, based on co-ocurrences in PubMed abstracts. The corpus is a KWIC (keyword in context) of English terms in PubMed articles, generated on-demand. The dictionary, the thesaurus, and the corpus can all be searched freely, without registration, but downloading them requires an extra license agreement. In addition, the Life Science Dictionary converted to a Resource Description Framework (RDF) format is maintained by the Database Center for Life Science (DBCLS, http://lsd.dbcls.jp/portal/), available under the Creative Commons Attribution-NoDerivs 3.0 Unported license (https://creativecommons.org/licenses/by-nd/3.0/).

The Interlinking Ontology for Biological Concepts (IOBC) [3] is a derivative of the JST-thesaurus (i.e., the thesaurus for indexing general science and technology publications used by the Japan Science and Technology Agency [JST]). This derivative was developed by the National Bioscience Database Center of JST, and is available from the NCBO BioPortal (https://bioportal.bioontology.org/ontologies/IOBC) under the Creative Commons Attribution-Non-Commercial 4.0 International license (https://creativecommons.org/licenses/by-nc/4.0/). This IOBC has 153,160 concepts, from the life sciences and related categories (such as chemistry), from the JST thesaurus 2015 version, written in both English and Japanese. Among them, 12,780 concepts also have corresponding MeSH UIDs. However, the MeSH information in the IOBC has not been updated from the one in the JST thesaurus 2015 version. The JST thesaurus itself is updated, and can be freely searched via web interface, although data download is not allowed. The JST also has the RDF version of the thesaurus, and its SPARQL endpoint was available to registered users of the service “J-Global Knowledge,” which unfortunately, has been discontinued.

Of these dictionaries, the JAMAS Thesaurus, JAMS Dictionary, and Life Science Dictionary are targeted to readers to seek information on already known terms, but cannot be searched by applications (such as morphological analysis), where the systems must find unspecified terms, over a broader context. Since creating derivatives of these dictionaries is not allowed for free, adapting them for NLP processes (such as morphological analysis), and especially making the results open, is very difficult. The IOBC does allow creation of derivatives, but only for non-commercial purposes, and its MeSH information is not updated, at least in the current version.

## Toward an Open Alternative of Japanese MeSH

In BLAH5, a small experiment was conducted as an attempt to map Japanese medical terms to MeSH, using open English-Japanese vocabularies, by first mapping Japanese terms to English terms, and the resulting English terms mapped to concepts in MeSH, which have the term as either its heading or one of its entry terms.

In our endeavor, we first used the English Wikipedia. From this, we retrieved Wikipedia entries (via DBpedia), with MeSH UIDs in the infobox, and Japanese language links were retrieved. The number of English Wikipedia entries with the MeSH UIDs was 2,719, of which 1,136 had links to Japanese Wikipedia entries. The number of distinct MeSH UIDs was 1,102, of which 1,075 were assigned to one Wikipedia entry. The remaining 27 UIDs were mapped to more than one term, but the relationships of the terms assigned the same UID were not always synonymous. Those other undesirable relationships included hypernym-hyponym (e.g., D003117 for *Dichromacy* and *Color blindness*; dichromacy is a type of color blindness) and cause-results (e.g., D006471), for *Gastrointestinal bleeding* and *Hematochezia*; hematochezia is a result of gastrointestinal bleeding). Further investigation is left for future work.

Second, use of open English-Japanese glossaries, for Japanese-English mapping and matching of English terms to the MeSH UID, was attempted. For this preliminary experiment, the MeSpEN English-Japanese glossary, a part of the multilingual medical glossary [4], developed by the Spanish National Cancer Research Center and the Barcelona Supercomputing Center (the version in this experiment was downloaded from http://temu.bsc.es/mespen/downloads/glossaries.tar.gz), was used. The English-Japanese glossary had 27,668 entries, although 18,325 unique English-Japanese ones were duplicate entries. Simple (lower-cased) matching against MH (heading) and MN (entry terms) of the MeSH 2018 descriptors, assigned the MeSH UIDs to 5,866 English-Japanese pairs. From this process, the number of unique MeSH UIDs assigned to these pairs was 2,955, and matched the remaining pairs to MeSH 2018 supplementary concepts (having assigned UIDs), leaving a remaining 1,725 pairs (1,173 unique UIDs).

## Conclusion

In this study, dictionaries for assigning MeSH information to Japanese medical terms was investigated. Although there are limited numbers of Japanese-English dictionaries that assign MeSH IDs (i.e., tree numbers) to Japanese terms, using them for NLP applications is not simple, due to license restrictions. An alternative approach, using open-source resources, was attempted, and yielded a partial success. Using other open resources, and qualitative evaluation of our results, will be the subject of future work.
